# Third contribution on Rovno amber silken fungus beetles: a new Eocene species of Cryptophagus (Coleoptera, Clavicornia, Cryptophagidae)

**DOI:** 10.3897/zookeys.130.1321

**Published:** 2011-09-24

**Authors:** G.Yu. Lyubarsky, E.E. Perkovsky

**Affiliations:** 1 Zoological Museum of Moscow State University, Bol’shaya Nikitskaya str. 6, Moscow, 103009 Russia; 2 Schmalhausen Institute of Zoology, National Academy of Sciences of Ukraine, Bogdan Khmelnitski str. 15, Kiev, 01601 Ukraine

**Keywords:** Cryptophagidae, *Cryptophagus*, Late Eocene, Rovno amber, Ukraine

## Abstract

*Cryptophagus alexagrestis* Lyubarsky & Perkovsky, **sp. n.** is described based on a fossil inclusion in Late Eocene Rovno amber (Ukraine). The new species is similar to the extant *Cryptophagus skalitzkyi* Reitter and *Cryptophagus dilutus* Reitter, differing from the latter by having a very transverse, short and dilated 10th antennal segment, and from the former by the very elongate segments of the flagellum.

## Introduction

The family Cryptophagidae is a group of small beetles with about 800 described species placed in approximately 50 genera and represented in all biogeographic realms. Most members of the family are free-living and mycophagous.

Silken fungus beetles are very common in the litter of forests in temperate climatic regions, where only Staphylinidae, Curculionidae and Carabidae are more abundant (Hyvärinen et al. 2006, Foottit and Adler 2009, Nitu et al. 2009, Šustek and Kristofik 2009). Most cryptophagids are nidicolous beetles; they are one of the most abundant beetles in the nests and burrows of rodents, birds and social insects (Lyubarsky 1996). On the other hand, in tropical Africa silken fungus beetles are rarely collected in litter (Kouadio et al. 2009). Cryptophagidae (together with Latridiidae) are fire-favoured insects (Muona and Rutanen 1994, Wikars and Schimmel 2001). Both adults and larvae of silken fungus beetles are commonly found on mold, fungi, under bark, as well as in decaying vegetation. Some genera are characterized by inquilinism with termites and social hymenopterans (Apidae, Vespidae and Formicidae) (Leschen 1999). Unlike many other groups of beetle symbionts, cryptophagid inquiline lineages do not have marked increases in speciation rates, despite the fact that the first records of some inquiline genera are from Late Eocene Baltic amber (Leschen 1999).

Discoveries of cryptophagids in fossil resins (see Lyubarsky and Perkovsky 2010) are of particular interest; over time, the generalization of such data can help to understand paleoclimates. Late Eocene Rovno amber represents a southern coeval analogue of the famous Baltic amber (Perkovsky et al. 2007, Perkovsky et al. 2010), collected in the northwest of Ukraine. The amber collection of the Schmalhausen Institute of Zoology of National Academy of Sciencesof Ukraine, Kiev (SIZK) contains more than 950 inclusions of beetles from unselected Rovno amber (Perkovsky et al. 2010; Kazantsev 2010), among them only three specimens are of silken fungus beetles (Lyubarsky and Perkovsky 2010; Lyubarsky and Perkovsky in press, this paper): one specimen of *Micrambe* Thomson, 1863 and two specimens of *Cryptophagus* Herbst, 1863. The amber piece containing the holotype of the new species described herein was mined in Pugach quarry (Klesov, north of Rovno region). Besides findings from fossil resins, Paleogene representatives of *Cryptophagus* are known as compression fossils from Argentina (*Cryptophagus suncholensis* Cockerell) and United States (*Cryptophagus bassleri* Wickham*, C. petricola* Wickham) (Ponomarenko and Kirejtshuk 2011).

The tarsal formula 5–5–5, 3-segmented antennal club, and closed procoxal cavities of the new species are quite characteristic of the family Cryptophagidae. The new species has antennal insertions exposed in dorsal view; pronotum with a well-developed marginal callosity; mesocoxal cavity closed laterally by the sternum; ventrite 1 longer than the remaining ventrites; and confused elytral punctation. These characters are indicative of the genus *Cryptophagus* (Cryptophaginae). Representatives of *Cryptophagus* are found in all biogeographic realms; the genus includes 137 species from the Palaearctic Region (Johnson et al. 2007).

Photographs were taken at the Paleontological Institute, Russian Academy of Sciences (Moscow) by V.A. Kolyada and the second author using a Leica M 165 microscope. To create diffused illumination, a cup of white styrofoam was placed between an object and a light source. The captured images were assembled with Helicon Focus 5.01 software.

## Taxonomy

**Family Cryptophagidae Kirby, 1837**

**Subfamily Cryptophaginae Kirby, 1837**

***Cryptophagus* Herbst, 1863**

### Cryptophagus alexagrestis

Lyubarsky & Perkovsky sp. n.

urn:lsid:zoobank.org:act:DB75F93F-056F-4B85-8505-5F255C694388

http://species-id.net/wiki/Cryptophagus_alexagrestis

Figs 1 2

#### Material.

 Holotype, SIZK K-24572, Klesov, Rovno amber, Late Eocene. Syninclusion: Chironomidae. Sex of the holotype unknown.

#### Etymology.

From Alex, in honour of Prof. Alexandr Rasnitsyn, and “agrestis”from Latin *ager* for field, farm.

#### Description.

 Body broadly elongate, slightly convex; head, pronotum, and elytra brown. Elytra slightly convex, covered with elevated pubescence.

Head transverse, of normal size, with hemispherical, somewhat coarsely facetted eyes, strongly and sparsely punctured. Antennae long, slender, with club reaching beyond base of pronotum, joints of flagellum elongate, 4th, 6th segment more than 1.5 times as long as broad, 5th 2 times as long as broad, 9th and 10th transverse, 11th obliquely oval, joints 9–11 equal in width.

Pronotum flat, not very strongly narrowed basally, distinctly transverse, barely 1.6 times broader than long, moderately not strongly and sparsely punctured (distance between punctures more than their diameter), an individual puncture less than the diameter of a facet. Pronotum without sublateral line, somewhat convex, sides narrowed basally and apically, with a single lateral tooth. Sides finely margined, anterior edge weakly sinuate. Callosity occupies at most one-seventh of side margin, with a small, elongate-oval patch of bare surface invisible from above; caudolateral corner obtuse angular, callosity without point. Lateral tooth far before middle of lateral margin. Posterior corners obtuse, base round, slightly sinuate, basal groove narrow.

Scutellum small, transverse. Elytra oval, humeral corners rounded, shoulders a little broader than maximum breadth of pronotum, 1.7 times as long as wide and 3.0 times as long as thorax, moderately convex, slightly flattened behind scutellum, with slightly rounded sides and a narrowly rounded apex, punctuation less strong and more sparse than that on pronotum.

Length of body 1.8 mm.

**Figure 1.**
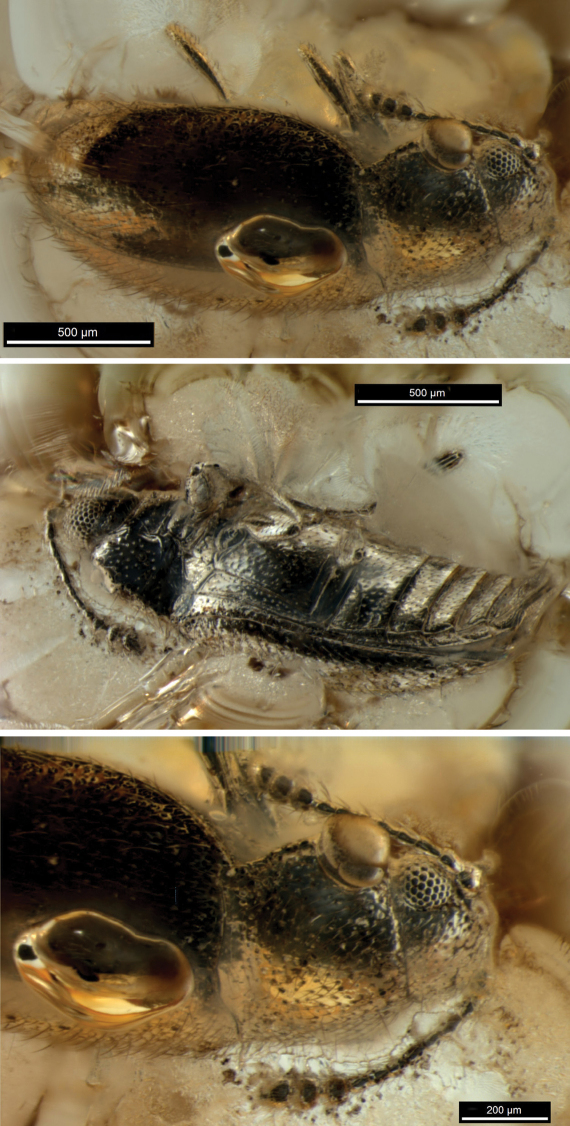
*Cryptophagus alexagrestis* sp. n., holotype (SIZK K-24572, Schmalhausen Institute of Zoology, Kiev) **a** body, dorsal **b** body, lateral **c** front part, dorsal.

**Figure 2.**
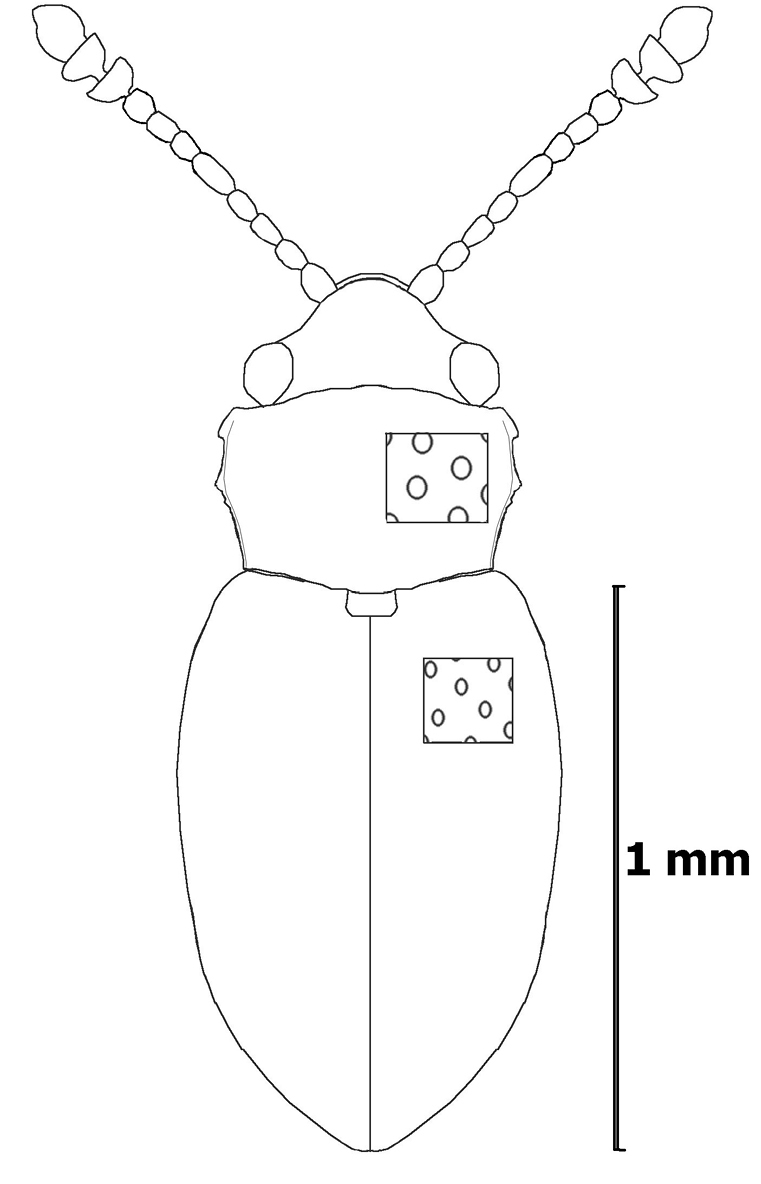
Dorsal view, *Cryptophagus alexagrestis* sp. n.

#### Remarks.

*Cryptophagus alexagrestis* sp. n.is most similar to the modern *Cryptophagus laterangulus* Reitter (Caucasus, Iraq, Iran, Turkmenistan, Kazakhstan), *Cryptophagus pseudoschmidti* Woodroffe (Eastern Europe, Siberia, Mongolia), *Cryptophagus dilutus* Reitter (Holarctic: North Africa, Europe, Caucasus, Middle Asia, Iran, Iraq, India, China, Siberia, North America), *Cryptophagus skalitzkyi* Reitter (Europe, Caucasus, Turkey, Iran, Turkmenistan, Uzbekistan, Tajikistan, Afghanistan, Pakistan, India, Kyrgyzstan, Kazakhstan, Eastern Siberia) with elevated elytral pubescence, bare surface of callosity not visible from above, 4th segment of antenna elongate, nearly 1.5 times as long as broad, lateral tooth far before middle of pronotum, small length of callosity (see Lyubarsky 2002). All mentioned modern species are widely distributed in steppe and desert zones, less common in the forest zone. *Cryptophagus dilutus* is common in the steppe and desert zones of Eurasia – in materials from Iraq, Iran, China, and Central Asia it is quite common. All mentioned species can be included in the key for identification of *Cryptophagus* (see Lyubarsky 2002, pp. 324–325, synthesis 17 and the following) with some changes, as shown below:

**Table:** 

1	Punctation of prothorax distinctly dense. Eyes often strongly prominent, hemispherical, with large facets. Lateral tooth extremely strongly prominent, but very small. Length 2.3–2.5 mm	*Cryptophagus laterangulus*
–	Punctation of prothorax sparse. Lateral tooth not extremely prominent	2
2	Eyes large, length greater than half the length of the head, asymmetrical conical, prominent, with large facets, diameter of facet more than 11 µm. 5th segment of antenna weakly elongated, 1.5 times as long as broad. Callosity occupies from 1/5 to 1/4 of length of lateral margin. Lateral tooth of prothorax normal or small, nearly reduced. Length 2.6–3.4 mm	*Cryptophagus pseudoschmidti*
–	Eyes normal in size, length less than half the length of the head, slightly prominent, with small facets, diameter of facet less than 11 µm. Lateral tooth of prothorax normal. Callosity occupies from 1/8 to 1/5 of length of lateral margin	3
3	10th segment of antenna rounded, about 1.5 times as broad as long. Callosity with point. Callosity occupies 1/6–1/5 length of lateral margin of pronotum. Length 2.1–2.7 mm	*Cryptophagus dilutus*
–	10th segment of antenna short and dilated, very transverse, as least twice as broad as long. Callosity short, its length occupying 1/8–1/6 length of lateral margin of pronotum	4
4	Facets of eyes small, diameter of facet less than 8 µm. Segments of flagellum weakly elongate, 4th, 5th, 6th segment 1.5 times as long as broad. Prothorax convex, very strongly narrowed basally. Punctuation of prothorax moderately dense, distance between neighbouring punctures equal to diameter of puncture or slightly less. Length 1.9–2.3 mm	*Cryptophagus skalitzkyi*
–	Facets of eyes large, diameter of facet more than 11 µm. Segments of flagellum strongly elongate, 4th, 6th segment more than 1.5 times as long as broad, 5th 2 times as long as broad. Prothorax flat, not very strongly narrowed basally. Length 1.8 mm	*Cryptophagus alexagrestis* sp. n.

## Supplementary Material

XML Treatment for Cryptophagus alexagrestis
